# Neurovestibular Dysfunction and Falls in Parkinson's Disease and Atypical Parkinsonism: A Prospective 1 Year Follow-Up Study

**DOI:** 10.3389/fneur.2020.580285

**Published:** 2020-10-29

**Authors:** Jeroen Venhovens, Jan Meulstee, Bas R. Bloem, Wim I. M. Verhagen

**Affiliations:** ^1^Department of Neurology and Clinical Neurophysiology, Canisius Wilhelmina Hospital, Nijmegen, Netherlands; ^2^Department of Neurology and Clinical Neurophysiology, Albert Schweitzer Hospital, Dordrecht, Netherlands; ^3^Department of Neurology, Radboud University Medical Centre, Donders Institute for Brain, Behaviour and Cognition, Nijmegen, Netherlands

**Keywords:** falls, Parkinson's disease, atypical Parkinsonism, follow-up, neurovestibular

## Abstract

Our primary aim was to determine whether neurovestibular laboratory tests can predict future falls in patients with either Parkinson's disease (PD) or atypical parkinsonism (AP). We included 25 healthy subjects, 30 PD patients (median Hoehn and Yahr stage 2.5, range 1–4), and 14 AP patients (6 multiple system atrophy, 3 progressive supranuclear palsy, and 5 vascular parkinsonism) in a case-control study design (all matched for age and gender). At baseline, all subjects underwent clinical neurological and neurotological assessments, cervical and ocular vestibular evoked myogenic potentials (VEMP), brainstem auditory evoked potentials (BAEP), subjective visual vertical measurements (SVV), and video nystagmography with caloric and rotary test stimulation. After 1 year follow-up, all subjects were contacted by telephone for an interview about their fall frequency (based upon fall diaries) and about their balance confidence (according to the ABC-16 questionnaire); only one participant was lost to follow-up (attrition bias of 1.4%). Cervical and ocular VEMPs combined with clinical tests for postural imbalance predicted future fall incidents in both PD and AP groups with a sensitivity of 100%. A positive predictive value of 68% was achieved, if only one VEMP test was abnormal, and of 83% when both VEMP tests were abnormal. The fall frequency at baseline and after 1 year was significantly higher and the balance confidence scale (ABC-16) was significantly lower in both the PD and AP groups compared to healthy controls. Therefore, VEMP testing can predict the risk of future fall incidents in PD and AP patients with postural imbalance.

## Introduction

Falls are highly prevalent in patients with Parkinson's disease (PD) or atypical parkinsonism (AP). Approximately 70% of PD patients have at least one fall episode annually (1). Fall incidents often lead to social isolation which may result in a reduced quality of life, because fall incidents can cause a fear of renewed fall episodes, possibly resulting in a self-imposed restriction of daily activities (2–4).

We previously showed that vestibular dysfunction is an independent risk factor for the occurrence of falls in PD and AP patients (4). The results of vestibular tests mainly reflect central neurological vestibular dysfunction, even though these patients usually do not complain of vertigo or dizziness (4). Patients with PD or AP who had experienced prior falls had more abnormal vestibular test results compared to non-falling patients. After exclusion of the well-established causes of falls (e.g., orthostatic hypotension, freezing of gait, cognitive problems and postural instability) 10–18% of the falling PD and AP patients had vestibular system abnormalities as the only identifiable cause for falling (4). We therefore concluded that vestibular system dysfunction, as established with neurovestibular laboratory tests, is an independent and relevant risk factor for falling in PD and AP.

The primary aim of this prospective study was to determine whether neurovestibular laboratory tests have predictive value for the occurrence of future falls in PD and AP patients and, if so, to determine their sensitivity, specificity, likelihood ratio's, and positive/negative predictive values. The secondary aim was to determine the fall frequency and balance confidence in both PD and AP after 1 year of follow-up, as compared with an age- and gender-matched healthy control group.

## Methodology

### Study Participants

Previously we described the methodology and baseline measurements of our study cohort in detail (4). Now we present the data after 1 year follow-up (median 12 months, range 12–14 months). Sixty-eight volunteers completed the follow-up study; 25 healthy controls (mean age 67, range 42–81, 15 men), 30 PD patients [mean age 70, range 59–81, 26 men, all fulfilling the UK Parkinson's Disease Society Brain Bank criteria (5), median Hoehn and Yahr stage 2.5, range 1–4], and 13 atypical parkinsonism (AP) patients (mean age 68, range 52–81, 8 men, 5 multiple system atrophy, 3 progressive supranuclear palsy, and 5 vascular parkinsonism, 1 patient with MSA-P was lost to follow-up). The MSA-P patients all fulfilled the diagnostic criteria for probable MSA-P as proposed in the consensus statement by Gilman (6). Supranuclear palsy patients (PSP) all fulfilled the NINDS-SPSP criteria for possible PSP (7). Vascular parkinsonism patients all fulfilled the criteria of the Winikates and Jankovic vascular rating scale (8).

The study was approved by the regional and local medical ethical committee (CMO Arnhem-Nijmegen, the Netherlands, number 2012/393) and was registered as well in the Dutch trial register (Nederlands Trial Register, NTR-3928). All volunteers signed an informed consent. Healthy controls and patients did not have a relevant medical history (i.e., no relevant neurological, otological, ophthalmological diseases, and/or absence of moderate-to-severe cognitive problems) with the exception of PD or AP (in combination with a related cerebrovascular disorder in the vascular parkinsonism group). Controls were matched for age and gender with the PD and AP patients. Sixty-nine volunteers were included in the baseline case-control study. Only one patient with multiple system atrophy with predominant parkinsonism (MSA-P) was lost to follow-up, resulting in an attrition bias of only 1.4%.

The participants were questioned about their medical history, medication, dizziness, gait and balance problems, prior falls and near falls, motor fluctuations, and freezing of gait. They underwent a detailed neurological and neurotological clinical examination with additional measurements for possible orthostatic hypotension (i.e., blood pressure measurement after lying supine for at least 15 min; followed by blood pressure measurements in a standing position after 1, 3, and 5 min). All PD and AP patients were tested during a regular medication on-state.

All participants completed: (a) the 16 items activities-specific balance confidence scale (ABC-16), (b) the dizziness handicap inventory (DHI), (c) the Edinburgh handedness inventory, (d) all subscales of the standardized unified Parkinson's disease rating scale (UPDRS), (e) the modified Hoehn and Yahr scale, (f) the Schwab and England activities of daily life (ADL) scale, and (g) a standardized falls questionnaire.

All participants received a Berg balance scale examination for quantitative balance assessment with additional pull-testing and functional reach testing for the assessment of the degree of postural imbalance. Partial postural imbalance was defined as a normal functional reach test in combination with an abnormal pull-test (i.e., sudden unexpected forceful backward shoulder pull without any specific prior instructions other than to remain standing upright; the patient was able to recover balance in more than two backward steps). Complete postural imbalance was defined as an abnormal pull-test: the patient would have fallen down if the examiner had not been present behind the patient to catch him/her during the fall. Patients that are informed in more detail about this test tend to shift their center of mass more anterior by leaning forwards in anticipation of the backward shoulder pull, which makes the test less reliable. For this reason patients were not informed in more detail prior to the test.

The neurovestibular laboratory tests conducted at baseline were: (a) cervical and ocular vestibular evoked myogenic potentials (VEMPs), (b) subjective visual vertical (SVV), and (c) video nystagmography (VNG) with additional caloric- and rotatory chair stimulation.

### Follow-Up by Telephone Interview

All participants were contacted by telephone for an interview 1 year after the baseline measurements. At baseline, they were instructed to keep record of their falls in the coming year. At the end of the baseline examinations, they were asked to keep track of their fall incidents during the following year by means of a fall diary. During the telephone interview, they were questioned about their fall frequency during the previous year, their fear of falling according to the ABC-16 questionnaire (16-items activities-specific balance confidence scale), acquired injuries related to fall incidents, and whether they had received medical treatment for such injuries.

### Statistical Analysis

The statistical database software SPSS version 23.0 (SPSS Inc., USA) was used for statistical analyses. The Shapiro-Wilks test was applied to determine whether parametrical tests were applicable (the null hypothesis, that the variable was distributed normally, was rejected when *p* ≤ 0.05). Because of this test result (Shapiro-Wilks *p*-values: 0.000 ≤ *p* ≤ 0.031, therefore the variables were not normally distributed) and due to the limited sample size of our study we had to apply non-parametrical tests for further statistical analyses. The Kruskal-Wallis one-way analysis by ranks test was applied for comparison of the continuous non-parametrically distributed data of the three independent groups (controls, PD, and AP), the Mann-Whitney-*U-*test for a group to group comparison, and a significance level of 5% was used for all analyses. An ordinal logistic regression analysis was applied for comparison of the categorical variables. We did not perform a multivariate regression analysis of the data due to the limited sample size. Also the sensitivity (number of true positives/(number of true positives + number of false negatives), specificity (number of true negatives/(number of true negatives + number of false positives), positive predictive value [PPV = number of true positives/(number of true positives + number of false positives)], negative predictive value [NPV = number of true negatives/(number of true negatives + number of false negatives)], positive likelihood ratio [sensitivity/(1 – specificity)], and the negative likelihood ratio [(1 – sensitivity)/specificity] were calculated for the different neurovestibular tests in relation to the future risk for falling in the different groups (patients with Parkinson's disease and atypical Parkinsonism).

## Results

We refer to Tables 1, 2 for the PD and AP patients' individual data concerning the clinical and neurovestibular neurophysiological baseline results. The individual 1-year follow-up results in relation to the baseline measurements are shown in Table 3, and we refer to Table 4 for the group characteristics. All four tables were adapted from our baseline study (4). Tables 5, 6 show the group characteristics concerning the difference between de falling and non-falling PD and AP patients in the cervical and ocular VEMP tests.

**Table 1:** Individual clinical characteristics of patients with Parkinson's disease and atypical Parkinsonism.

		**ID**	**Age**	M/F^*^	Disease duration^**^	**Dominant side**	**Hoehn-Yahr stage**	Ortho^***^	FOG^****^	PI^*****^
Parkinson's disease		1	68	M	4.0	Right	2	–	–	–
		2	60	M	2.0	Left	2.5	+	+	C
		3	73	M	7.0	Symmetrical	3	+	–	C
		4	60	M	2.0	Left	1.5	–	–	P
		5	78	M	2.0	Left	1.5	+	–	P
		6	73	M	2.0	Right	3	–	–	C
		7	64	M	2.0	Right	1	–	–	–
		8	59	F	4.0	Right	2	–	–	–
		9	72	M	5.5	Right	2.5	–	–	P
		10	80	M	1.5	Right	2	–	–	–
		11	58	M	4.0	Left	1	–	–	P
		12	66	M	4.5	Right	3	–	–	C
		13	66	M	2.0	Right	1	–	–	–
		14	70	M	5.0	Left	2.5	–	–	P
		15	59	M	12.0	Left	2	–	–	P
		16	75	F	10.0	Right	3	–	–	C
		17	75	M	3.0	Right	2.5	–	–	P
		18	59	M	2.0	Left	2	–	–	–
		19	76	F	3.5	Right	2.5	–	–	P
		20	76	M	6.0	Left	3	–	+	C
		21	81	F	22.0	Symmetrical	4	–	+	C
		22	75	M	8.0	Left	3	–	+	C
		23	67	M	3.0	Right	2.5	+	–	P
		24	71	M	5.0	Symmetrical	2	–	–	–
		25	76	M	8.0	Left	2.5	–	+	P
		26	65	M	6.0	Right	2	–	–	P
		27	76	M	2.0	Right	1.5	+	–	–
		28	69	M	3.0	Left	2.5	–	–	P
		29	78	M	12.0	Left	2.5	+	+	P
		30	65	M	2.0	Symmetrical	2	–	–	–
Atypical Parkinsonism	MSA	31	73	F	2.5	Symmetrical	4	+	–	C
		32	67	F	6.0	Symmetrical	3	+	–	C
		33	69	M	3.0	Symmetrical	3	+	+	C
		34	71	M	4.0	Symmetrical	4	+	+	C
		35	57	M	9.5	Right	2	+	–	–
		36	61	M	5.0	Symmetrical	4	+	–	C
	PSP	37	71	F	1.5	Right	1	–	–	P
		38	61	M	2.0	Symmetrical	3	–	–	C
		39	74	M	3.0	Symmetrical	3	–	–	P
	Vascular	40	71	F	2.0	Left	2.5	+	–	C
		41	52	F	3.5	Right	2	–	–	–
		42	76	M	6.5	Symmetrical	2.5	–	–	P
		43	65	M	1.0	Left	3	–	–	P
		44	81	M	3.0	Symmetrical	3	–	–	C

*Adapted from Venhovens et al. (4)*.

* *Gender (male or female)*.

** *Disease duration (calculated from symptom onset in years)*.

*** *Orthostatic hypotension (Ortho)*.

**** *Freezing of gait (FOG)*.

***** *Postural imbalance, PI (C, complete imbalance on pull testing without unaided recovery of balance; P, partial imbalance on pull testing with unaided recovery of balance requiring 2 or more backward steps)*.

**Table 2:** Baseline individual test results of patients with Parkinson's disease and atypical Parkinsonism.

		**ID**	cVEMP^*^	oVEMP^*^	SVV^**^	**VNG** **+** calorisation^***^						
						**C**	**S**	**O**	**P**	**N**	**R**	**F**
Parkinson's disease		1	–/–	–/A	+	–	–	–	–	–	–	–
		2	–/–	–/–	+	VP(r)	–	–	–	–	–	–
		3	–/–	A/A	–	VP(l)	–	+	+	–	–	–
		4	–/–	–/–	–	–	–	–	–	–	–	–
		5	–/–	–/–	–	–	–	–	–	–	–	–
		6	–/–	–/A	–	–	+	–	+	–	–	–
		7	–/D	–/–	–	–	–	–	–	–	–	–
		8	A/–	–/D	–	–	–	–	–	–	–	–
		9	–/–	–/–	–	–	–	–	+	–	–	–
		10	D/–	D/–	+	–	–	–	+	–	+	–
		11	D/D	–/A	–	–	–	–	–	–	–	–
		12	–/–	D/A	–	VP(l)	+	+	+	–	+	+
		13	–/–	–/D	–	–	–	–	–	–	–	–
		14	–/–	–/–	–	–	–	–	+	–	+	–
		15	–/–	–/D	–	–	–	–	–	–	–	–
		16	D/–	–/A	+	–	–	–	+	–	–	–
		17	–/–	–/–	+	–	+	+	+	–	–	–
		18	–/–	–/–	–	–	+	–	+	–	+	–
		19	–/–	–/D	+	–	+	–	+	–	+	–
		20	–/–	–/A	+	–	+	–	+	–	–	–
		21	–/–	D/A	–	–	–	–	+	–	–	+
		22	–/D	D/A	–	–	+	–	+	–	–	–
		23	–/–	–/A	–	–	–	–	–	–	–	–
		24	–/D	–/D	+	–	+	–	–	–	–	–
		25	D/–	–/–	+	–	+	+	+	–	–	–
		26	–/D	–/–	–	–	+	–	–	–	–	–
		27	–/–	–/–	+	–	–	–	+	–	–	–
		28	–/–	–/–	+	VP(b)	+	–	+	–	+	–
		29	A/D	–/–	–	–	+	+	–	–	–	–
		30	A/–	–/–	+	–	+	–	+	–	–	–
Atypical Parkinsonism	MSA	31	–/–	A/A	–	–	+	+	+	–	–	+
		32	D/–	–/A	+	–	+	+	+	–	–	+
		33	–/–	–/A	–	–	+	+	+	–	+	–
		34	A/–	–/–	–	DP(l)	+	–	+	–	+	–
		35	–/D	–/A	–	–	+	+	+	+	–	+
		36	D/D	–/–	–	–	+	+	+	–	–	+
	PSP	37	D/–	–/–	+	–	+	–	+	–	–	–
		38	–/–	–/–	–	–	+	–	–	–	–	–
		39	–/–	–/–	–	–	+	–	+	–	+	–
	Vascular	40	D/–	D/–	+	–	–	–	–	–	–	–
		41	–/–	–/A	–	VP(r)	–	+	+	–	+	+
		42	–/–	–/–	+	–	+	–	+	–	–	–
		43	–/–	–/A	–	VP(r)	+	–	+	–	–	–
		44	D/D	D/–	–	–	–	–	–	–	–	–

Adapted from Venhovens et al. (4).

* Cervical (cVEMP) and Ocular (oVEMP) vestibular evoked myogenic potentials: Right/Left responses (A, absent response; D, delayed response; –, normal response).

** Subjective Visual Vertical (SVV): +, abnormal; –, normal.

*** Videonystagmography (VNG) and calorisation results: C[alorisation; VP, vestibular paresis (left/right/bilateral); DP, directional preponderance (left/right/bilateral)]; S(accade testing); O(ptokinetics); smooth P(ursuit); spontaneous N(ystagmus) in dark and light conditions; R(otary) chair testing; F(ixation) suppression testing; +, abnormal; –, normal.

**Table 3:** Individual clinical characteristics concerning the fall frequencies and balance confidence at baseline and during follow-up of patients with Parkinson's disease and atypical Parkinsonism.

		**ID**	Falls baseline^*^	Falls follow-up^*^	ABC-16 baseline^**^	ABC-16 follow-up^**^
Parkinson's disease		1	–	–	72	57
		2	–	–	99	100
		3	Y	–	91	90
		4	–	–	95	80
		5	–	–	73	76
		6	–	–	70	64
		7	–	–	78	74
		8	–	–	78	68
		9	–	–	86	90
		10	6M	–	64	59
		11	3M	3M	73	65
		12	–	–	72	65
		13	–	–	98	99
		14	–	–	61	64
		15	–	–	58	71
		16	1M	1M	51	31
		17	–	–	68	63
		18	–	–	71	73
		19	–	6M	38	36
		20	W	W	59	54
		21	W	W	70	66
		22	1M	W	48	46
		23	6M	–	99	95
		24	–	–	76	61
		25	Y	3M	75	68
		26	–	–	74	75
		27	–	–	68	70
		28	–	–	97	93
		29	W	W	58	82
		30	Y	–	68	80
Atypical Parkinsonism	MSA	31	1M	1M	40	20
		32	3M	1M	58	45
		33	W	D	46	0
		34	W	D	39	29
		35	–	–	62	57
		36#	3M	#	43	#
	PSP	37	6M	W	76	58
		38	–	–	75	79
		39	–	–	64	43
	Vascular	40	1M	1M	57	59
		41	6M	–	98	96
		42	Y	–	79	80
		43	6M	3M	92	71
		44	–	–	55	28

Adapted from Venhovens et al. (4).

* Frequency of falling (Y, once a year; 6M, once every 6 months; 3M, once every 3 months; 1M, monthly; W, weekly; D, daily).

** *ABC-16 questionnaire (16-items specific confidence of balance scale). ^#^Loss to follow-up*.

**Table 4:** Group characteristics and comparison between the groups concerning the different test results in the Parkinson's disease, atypical Parkinsonism, and healthy control groups.

	**Parkinson's disease**	**Atypical Parkinsonism**	**Healthy controles**	*P*-value^*^	*P*-value group-to-group comparison^*^
**Number of subjects (*****N*****)**	30	14^**^	25	0.054	–
**Number of falling patients:**					
- Baseline (N, percentage) - Follow-up (N, percentage)	11 (37) 8 (27)	10 (71) 7 (54)	3 (12) 5 (20)	** <0.001** **0.043**	**Baseline P**_**PD−controls**_ **=** **0.037** **Baseline P**_**AP−controls**_ ** <0.001** **Baseline P**_**PD−PA**_ **=** **0.032** Follow-up P_PD−controls_ = 0.571 **Follow-up P**_**AP−controls**_ **=** **0.034** Follow-up P_PD−PA_ = 0.090
**Average falls/year (baseline):**					
- All patients in total (average, SD) - Only falling patients (average, SD)	6.4 (15.8) 16.0 (22.1)	10.2 (18.1) 14.3 (20.3)	0.3 (0.9) 2.7 (1.2)	** <0.001**	**P**_**PD−controls**_ **=** **0.035** **P**_**AP−controls**_ **=** **0.001** **P**_**PD−PA**_ **=** **0.039**
**Average falls/year (follow-up):**					
- All patients in total (average. SD) - Only falling patients (average, SD)	7.7 (3.3) 28.8 (8.8)	63.2 (37.4) 117.4 (64.2)	0.9 (0.5) 4.4 (2.0)	**0.032**	P_PD−controls_ = 0.394 **P**_**AP−controls**_ **=** **0.032** P_PD−PA_ = 0.116
**Change in falls/year (absolute, percentage)**					
- All patients in total (absolute, percentage) - Only falling patients (absolute, percentage)	+1.3 (+21.1) +9.8 (+51.3)	+53.0 (+518.7) +103.1 (+721.0)	+0.6 (+175.0) +1.7 (+65.0)	0.164	–
**ABC-16 fear of falling, baseline:**					
- All patients in total (average, SD) - Only falling patients (average, SD) - Only non-falling patients (average, SD)	72.9 (2.8) 59.0 (4.6) 78.0 (2.8)	64.7 (5.1) 57.9 (6.4) 72.2 (6.3)	82.3 (3.6) 60.8 (9.4) 87.7 (3.0)	**0.006** 0.914 **0.010**	**All patients P**_**PD−controls**_ **=** **0.028** **All patients P**_**AP−controls**_ **=** **0.004** All patients P_PD−AP_ = 0.096 **Non-falling P**_**PD−controls**_ **=** **0.014** **Non-falling P**_**AP−controls**_ **=** **0.009** Non-falling P_PD−AP_ = 0.116
**ABC-16 fear of falling, follow-up:**					
- All patients in total (average, SD) - Only falling patients (average, SD) - Only non-falling patients (average, SD)	70.5 (3.1) 56.0 (6.2) 75.8 (2.9)	51.2 (7.5) 38.8 (8.4) 63.8 (10.5)	80.3 (3.6) 53.8 (7.9) 87.0 (2.4)	**0.001** 0.190 **0.009**	**All patients P**_**PD−controls**_ **=** **0.022** **All patients P**_**AP−controls**_ **=** **0.001** **All patients P**_**PD−AP**_ **=** **0.018** **Non-falling P**_**PD−controls**_ **=** **0.010** **Non-falling P**_**AP−controls**_ **=** **0.004** Non-falling P_PD−AP_ = 0.081
**Change in ABC-16:**					
- All patients in total (absolute, percentage) - Only falling patients (absolute, percentage) - Only non-falling patients (absolute, percentage)	−2.4 (−3.3) −3.0 (−5.1) −2.2 (−2.8)	−13.5 (−20.9) −19.1 (−33.0) −8.4 (−11.6)	−2.0 (−2.4) −7.0 (−11.5) −0.7 (−0.8)	**0.028** 0.156 0.506	**All patients P**_**PD−controls**_ **=** **0.022** **All patients P**_**AP−controls**_ **=** **0.001** **All patients P**_**PD−AP**_ **=** **0.018**
**Fall injury, baseline (*****N*****, percentage):**					
- No injury - Minor (e.g., cuts and bruises) - Intermediate (e.g., simple fractures) - Severe (e.g., fractures requiring surgery)	1 (9) 9 (82) 1 (9) 0 (0)	1 (10) 8 (80) 0 (0)1 (10)	0 (0) 1 (33) 0 (0)	0.714	–
**Fall injury, follow-up (*****N*****, percentage):**					
- No injury - Minor (e.g., cuts and bruises) - Intermediate (e.g., simple fractures) - Severe (e.g., fractures requiring surgery)	0 (0) 7 (88) 0 (0) 1 (12)	0 (0) 6(86) 1 (14) 0 (0)	1 (20) 3(60)	0.414	–
**Treatment, baseline (*****N*****, percentage):**					
- No treatment necessary - Self-treatment - Outpatient doctor's treatment - Hospital admission (no surgery) - Hospital admission for surgery	2 (18) 6 (55) 2 (18) 1 (9) 0 (0)	1 (10) 7 (70) 1 (10) 0 (0) 1 (10)	0 (0) 2 (67) 1 (33) 0 (0) 0 (0)	0.714	–
**Treatment, follow-up (*****N*****, percentage):**					
- No treatment necessary - Self-treatment - Outpatient doctor's treatment - Hospital admission (no surgery) - Hospital admission for surgery	0 (0) 7 (88) 0 (0) 0 (0) 1 (12)	0 (0) 5 (72) 1 (14) 1 (14) 0 (0)	1 (20) 2 (40) 1 (20) 0 (0) 1 (20)	0.427	–

Adapted from Venhovens et al. (4).

* The P-value is calculated by means of an ordinal regression calculation (in the categorical variables), the Kruskal-Wallis one-way analysis of variance by ranks test (in the continuously distributed, independent, and non-parametrical variables for comparison of the 3 groups), and the Mann-Whitney-U test (in the continuously distributed, independent, and non-parametrical variables for group to group comparison). A significance level of 5 percent (i.e., P ≤ 0.05) was adopted for each analysis and significant P-value results are printed in bold.

** Fourteen patients completed the baseline examinations and one patient was lost to follow-up.

**Table 5:** Group characteristics concerning the falling and non-falling Parkinson and atypical Parkinsonism patients in the cervical and ocular vestibular evoked myogenic potentials tests.

	**Cervical VEMP**		**Ocular VEMP**		cVEMP and/or oVEMP combined^*^		cVEMP and oVEMP combined^**^	
	**Abnormal**	**Normal**	**Abnormal**	**Normal**	**Abnormal**	**Normal**	**Abnormal**	**Normal**
Falling patients (absolute number)^***^	9	6	11	4	15	0	5	10
Non-falling patients (absolute number)^***^	8	20	13	15	16	12	5	23
All patients combined (absolute number)^#^	17	26	24	19	31	12	10	33

* Abnormal result was defined as having at least one abnormal cVEMP and/or oVEMP test.

** Abnormal result was defined as having an abnormal test result in both cVEMP and oVEMP tests combined.

*** Absolute number of patients that did fall or did not fall during follow-up. ^#^One patient was lost to follow-up, therefor the total group of patients studied was 43.

**Table 6:** Group characteristics concerning a selected group of falling and non-falling Parkinson and atypical Parkinsonism patients, with postural instability on pull-testing, in the cervical and ocular vestibular evoked myogenic potentials tests.

	cVEMP and/or oVEMP combined in patients with postural instability^*^		cVEMP and oVEMP combined in patients with postural instability^**^	
	**Abnormal**	**Normal**	**Abnormal**	**Normal**
Falling patients (absolute number)^***^	15	0	5	10
Non-falling patients (absolute number)^***^	7	10	1	16
All patients combined (absolute number)^#^	22	10	6	26

* Abnormal result was defined as having at least one abnormal cVEMP and/or oVEMP test.

** Abnormal result was defined as having an abnormal test result in both cVEMP and oVEMP tests combined.

*** Absolute number patients that did fall of did not fall during follow-up. ^#^One patient was lost to follow-up, therefor the total group of patients studied was 43.

From our data in Table 4 and additional group-to-group comparisons it may be inferred that the number of falling PD and AP patients was statistically significantly higher in comparison to age- and gender-matched healthy control subjects at baseline. However, at follow-up 1-year later only AP patients fell statistically significantly more often. Moreover, the percentage of falling AP patients was higher than the PD patients, however the difference was only statistically significant at baseline.

The PD patients and the AP patients have a statistically significantly higher fall frequency at baseline in comparison to healthy controls, however during follow-up only the difference between the AP patients and healthy controls remained statistically significant.

The 16-items activities-specific balance confidence scale (ABC-16) differed significantly between the groups in total and the non-falling patients both at baseline and during follow-up 1 year later, which showed a higher fear of falling in the PD patients and especially the AP patients in comparison to the healthy controls. The fear of falling in only the falling patients, however, was the same across the groups. The change in ABC-16 scores between the baseline measurements and after 1 year follow-up was statistically significant in the total group, which showed a larger increase concerning the fear of falling in de PD and AP patients in comparison to the healthy controls. Moreover, the AP patients also had a larger increase concerning the fear of falling in comparison to the PD patients during follow-up 1 year later. The fall related injuries both at baseline or during follow-up and their treatments did not differ significantly.

## Discussion

Laboratory examinations, and especially the vestibular evoked myogenic measurements, VEMPs (an abnormal VEMP result, defined as having at least one abnormal result at both the cervical and/or ocular VEMP tests combined) have a sensitivity of 100% to predict the occurrence of falls (all 15 falling patients had abnormal test results, see Table 5), at the cost of a low PPV of 48.4% (15 of the 31 patients will fall during follow-up). The specificity is 42.9% (12 of 28 the non-falling PD and AP patients have normal cervical and ocular VEMP results, however also 16 of these 28 non-falling patients have abnormal test results). The NPV when both the ocular and cervical VEMP tests are normal is 100% (none of the 12 patients with normal test results will fall during a 1-year follow-up). The positive likelihood ratio for falling when at least one cervical and/or ocular VEMP test is abnormal is 0.9 (15 patients out of the 31 patients with an abnormal result will fall and the other 16 patients with abnormal test results will not fall during a 1-year follow-up), and 1.0 when both VEMP tests are abnormal. The negative likelihood ratio for falling when both the cervical and ocular VEMP tests are normal is 0 (none of the patients that will fall had normal results and 12 of the non-falling patients had normal results), and 0.4 when the cervical and/or ocular VEMP test was abnormal. Therefore, in our pilot study normal cervical and ocular VEMP results in AP and PD patients have a very high negative predictive value for falling in the following year, which means that these patients have a very low risk for falling in the coming year. However, these tests have a very limited diagnostical usefulness to detect those PD and AP patients ad-risk for falling.

The presence of freezing of gait is also a strong predictor for the occurrence of falls (seven out of eight patients will fall, yielding a PPV of 87.5%). However, the sensitivity for detecting patients at risk for future falls is limited as only seven out of 15 patients will be detected (46.6%). The sensitivity for detecting patients at risk for falling is very high concerning the clinical testing for the presence of postural instability (100%; i.e., all 15 falling patients had postural instability), but the PPV is only 46.9% (only 15 of the 32 patients with abnormal test results will fall during 1-year follow-up).

Therefore, there is no single clinical or laboratory test (that is independent of clinical tests) with a high positive predictive value, high likelihood ratio, and a high sensitivity for detecting patients with a high risk for falling. These three test characteristics are needed to make a screening test that is useful in clinical practice in order to detect all patients at risk, however to also prevent a large number of false positive results which would lower the diagnostic value.

However, when both tests are used in combination (i.e., to use VEMP testing for additional screening for future fall incidents in those patients who at least have partial postural imbalance) the sensitivity will still remain 100% (15 out of the 15 falling patients will be detected, see Table 6); and the PPV will subsequently be 68.2% when only the ocular and/or the cervical VEMP is abnormal (15 out of the 22 patients with positive results will fall during the year follow-up), or 83.3% when both VEMP tests are abnormal (5 out of 6 patients with positive results will fall during the follow-up year; with 5 out of 15 falling patients having abnormal results in both VEMP tests). Respectively, this results in positive likelihood ratio's of 2.1 (when only the cervical and/or ocular VEMP tests is abnormal) or 5.0 (when both the cervical and ocular VEMP tests are abnormal). The NPV will subsequently be 61.5% (when only the cervical and/or ocular VEMP tests is normal) and 100% (when both the cervical and ocular VEMP tests are normal), respectively, resulting in negative likelihood ratio's of 0.6 and 0. Therefore, we conclude that (ab)normal cervical and/or ocular VEMP tests in a selected group op PD and AP patients (i.e., with postural instability) is most useful to detect patients ad-risk for falling and to identify patients with a very low risk for falling.

Cervical and ocular VEMP testing combined with the clinical evaluation for postural instability gives additional information concerning the future fall risk of PD and AP patients compared to clinical evaluation of postural instability alone. However, the presence of freezing of gait is such a strong predictor for future falls in both PD and AP patients (PPV 87.5%; all the falling patients with freezing of gait also had abnormal VEMPs and postural instability) that VEMP testing in these patients does not have any additional value. Therefore, cervical and ocular VEMP testing seems to give additional information concerning the future fall risk in selected PD and AP patients (those patients who have postural instability in the absence of freezing of gait). However, one could speculate whether this additional information (possible increase of the PPV from 46.9% to 68.2–83.3%) will aid in guiding future fall prevention therapies (for instance through physical therapy) as PD and AP patients with postural imbalance already have a high risk for falling. A practical consensus-based overview concerning the risk factors and management of falls in PD was published emphasizing the multifaceted origin of the falls and the need for a personalized approach (9).

Decreasing the risk of falling is important, as fall incidents will result in a lowered subjective balance confidence (as can be concluded from the data in Table 4), secondarily resulting in self-imposed restrictions in daily life, ultimately leading to social isolation (2, 3). Moreover, patients with parkinsonism are more at risk for fall-related injuries, such as hip fractures secondarily leading to a higher morbidity, mortality, and health care costs in comparison to individuals without Parkinsonism (3, 10).

Our study also has some important limitations, which we also mentioned earlier (4). The first limitation relates to the small sample size; this was explained by the strict inclusion/exclusion criteria and the lengthy nature of the neurovestibular testing, which is especially demanding for elderly and AP patients. Therefore, the results from this study need to be interpreted cautiously and as hypothesis-generating for further research, especially in the heterogeneous AP group. This study, to our knowledge, is the first (prospective) study ever conducted to assess whether neurovestibular tests in both PD and AP can predict future fall incidents. Seen as this pilot study offers the first insights in the prediction of future fall incidents, especially in the heterogeneous AP group, we decided to use the data from both the AP and PD groups for further statistical analysis as discussed above. However, due to the limitations mentioned above, we advocate further research concerning the additional value of VEMP testing for predicting the risk of falling in larger PD and AP (sub)groups to confirm our findings. The second limitation is, that the volunteers may have had a bias concerning the recollection of their falling incidents. We tried to overcome this issue by asking the volunteers to keep track of their fall incidents by keeping personalized fall diaries. However, mal-compliance could bias the results possibly leading to an underestimation of the true fall incidence. Therefore, we questioned the volunteers about their fall frequency instead of the absolute number of falls, to minimize the effects of mal-compliance and recollection bias.

To conclude, we found a high prevalence in the number of falling patients and fall incidents in our follow-up study after 1 year in both PD and AP patients. After 1 year, especially the frequency of the fall incidents in the AP group increased in comparison to the PD and control group, which was not statistically significant (probably as a result of the small group size) as the other groups did also show a less pronounced increase in the number of fall incidents. The risk of future falls in PD and AP patients can be predicted better when patients with postural imbalance on clinical testing are additionally tested by means of cervical and ocular VEMP testing (with the exclusion of patients with freezing of gait). However, it remains unclear if the increase in future fall risk (possible PPV increase from 46.9% to 68.2–83.3%) will aid in the different utilization of fall prevention strategies as PD and AP patients with postural imbalance already have a high risk of falling.

## Data Availability Statement

The raw data supporting the conclusions of this article will be made available by the authors, without undue reservation.

## Ethics Statement

The studies involving human participants were reviewed and approved by the regional and local medical ethical committee (CMO Arnhem-Nijmegen, the Netherlands, number 2012/393). The patients/participants provided their written informed consent to participate in this study.

## Author Contributions

JV, WV, JM, and BB: conception or design of the work. JV: data collection. JV, JM, and WV: data analysis and interpretation. JV: drafting the article: first version. JV, WV, JM, and BB: drafting the article: second and third versions. WV, JM, and BB: critical revision of the article. JV, WV, JM, and BB: final approval of the version to be published. All authors have read the final manuscript and have agreed with this submission.

## Conflict of Interest

The authors declare that the research was conducted in the absence of any commercial or financial relationships that could be construed as a potential conflict of interest.
